# Supplementary material for: Environmental and molecular noise buffering by the cyanobacterial clock in individual cells
Source: Nat Commun. 2025 Apr 15;16:3566. doi: 10.1038/s41467-025-58169-8 (PMC12000584; doi:10.1038/s41467-025-58169-8)
Supplement: Supplementary file 2 — Description of Additional Supplementary Files [file 41467_2025_58169_MOESM2_ESM.pdf]

Description of Additional Supplementary Files:

Supplementary Data 1: Plasmids and primers used in this study.

Supplementary Movie 1: A time-lapse movie of a WT strain (from a data set shown in Fig. 2). The strain expresses a *pkaiBC:eYFP:fsLVA* transcriptional reporter (shown in green), which displays circadian oscillations. Cells are entrained in the chip upon exposure to 12 h darkness and then observed under free-running, continuous light conditions (medium LL, 20 μmol m^{-2} s^{-1}). Phase contrast images are shown in the background in grey. The imaging frequency is every 45 min.

Supplementary Movie 2: A time-lapse movie of a WT strain (from a data set shown in Fig. 4). The strain expresses a *pkaiBC:eYFP:fsLVA* transcriptional reporter (shown in green), which displays circadian oscillations. Cells are entrained in the chip upon exposure to 12 h darkness and then observed under square-shaped 12 h: 12 h square LD cycles (medium LD, 24 μmol m^{-2} s^{-1}). Phase contrast images are shown in the background in grey. The imaging frequency is every 60 min during the day and every 240 min during the night.
